# Geographic and racial/ethnic patterns of polysomnography use among children enrolled in Medicaid, 2017–2019

**DOI:** 10.1007/s44470-026-00149-w

**Published:** 2026-08-03

**Authors:** Colleen C. McLaughlin, Jesse L. Hawke, Emily F. Boss, Guy M. Brock, Meredith M. Lind, Tasleem J. Padamsee, Prasanth Pattisapu, Deena J. Chisolm, Laura J. Chavez

**Affiliations:** 1 Center for Child Health Equity and Outcomes Research, Abigail Wexner Research Institute at Nationwide Children’s Hospital, 700 Children’s Drive, Columbus, OH USA; 2 Department of Otolaryngology, Johns Hopkins University School of Medicine, 601 N. Caroline Street, Baltimore, MD USA; 3 Department of Biomedical Informatics and Center for Biostatistics, Ohio State University, 250 Cunz Hall, 1841 Neil Ave, Columbus, OH USA; 4 Department of Otolaryngology, Ohio State University and Nationwide Children’s Hospital, 700 Children’s Drive, Columbus, OH USA; 5 Division of Health Services Management and Policy, College of Public Health, Ohio State University, 280 F Cunz Hall, 1841 Neil Ave, Columbus, OH USA; 6 Department of Pediatrics, College of Medicine, Ohio State University, 700 Children’s Drive, Columbus, OH USA

**Keywords:** Polysomnography, Pediatrics, USA, Geographic mapping, Neighborhood racial ethnic composition, Urban–rural

## Abstract

**Purpose:**

The purpose of this cross-sectional observational study was to describe the geographic distribution of utilization of polysomnography (PSG) among children enrolled in Medicaid/Child Health Insurance Program from 2017 to 2019.

**Methods:**

The data source was the Transformed Medicaid Information System (T-MSIS) research analytic files. PSG among children ages 0 to 18 years was identified from the claims data. All children enrolled in Medicaid with at least one service utilization claim formed the denominators for calculation of age-adjusted rates per 10,000 children at the US Census Bureau Division, state and local levels. Geographic patterns were visualized with maps and plots. PSG rates were modeled by Rural–Urban Commuting Area (RUCA) classification and race-ethnicity composition at the ZIP code level. Data quality concerns resulted in exclusion of Rhode Island and Vermont.

**Results:**

There were 478,568 PSGs identified among eligible children in the claims data, resulting in a national rate of 50.1 PSGs per 10,000 Medicaid enrolled children per year. There was a fourfold difference in rates at the state level, ranging from 23 PSGs per 10,000 person-years (Kansas) to 93 per 10,000 (Michigan). New England and the Great Lakes regions had the highest local levels, while the Pacific and West South Central divisions had the lowest. ZIP codes with higher percent White population (> 90%) and those in metropolitan areas had the highest PSG rates.

**Conclusion:**

This descriptive study of pediatric PSG utilization in the USA can inform efforts to provide more equitable access to PSG and refinement of practice guidelines for referral to PSG.

**Brief Summary:**

**Current knowledge/study rationale:**

Polysomnography provides a definitive diagnosis for obstructive sleep apnea and other sleep problems. Although there are concerns about variation in access, the utilization patterns of pediatric polysomnography in the USA is unknown.

**Study impact:**

At the state level, polysomnography among children enrolled in Medicaid exhibited a fourfold difference in rates. ZIP codes in metropolitan areas and those with a high proportion of White non-Hispanic population had the highest utilization rates. These data can inform consensus among professional groups and healthcare policy researchers to improve equitable utilization.

**Graphical Abstract:**

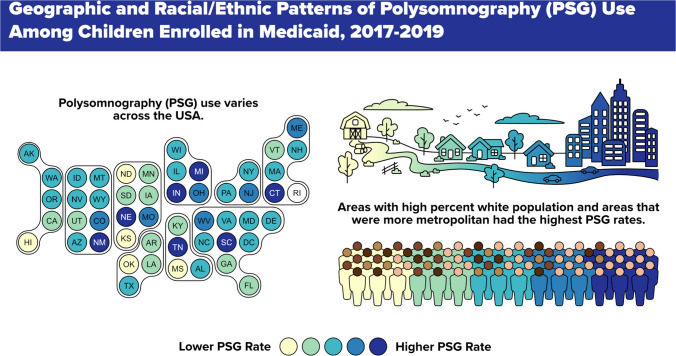

**Supplementary Information:**

The online version contains supplementary material available at 10.1007/s44470-026-00149-w.

## Introduction

Polysomnography (PSG) is used among children for the assessment of a wide range of sleep conditions, the most common of which is obstructive sleep-disordered breathing (oSDB) [1, 2]. Polysomnography provides a definitive diagnosis of obstructive sleep apnea (OSA), informs decision-making when the need for tonsillectomy is uncertain, and allows an assessment of perioperative breathing risks [3]. Polysomnography is also used to assist in the diagnosis of other sleep conditions, such as sleep movement disorders and parasomnias [1].

Geographic variation of PSG use in children is not well understood. Although the potential for geographic variation in access to pediatric PSG in the USA has been discussed, there is very limited empirical data [3–5]. There are a handful of studies addressing geographic access to general sleep medicine, yet none have focused on children [6–9]. Children make up only a small proportion of patients undergoing PSG, and access barriers for children are likely to differ from those of adults. Importantly, PSG in children uses both unique equipment and measurement algorithms compared to adult studies and therefore requires specially equipped sleep labs for access. Home sleep studies are not approved for use in children. Given these unique considerations in pediatrics, updated information on the geography of PSG use for children is needed.

This study fills a gap in our understanding of the variation of pediatric PSG use in the USA for children. We utilize administrative claims data from Medicaid and the Child Health Insurance Program (CHIP) from 2017 to 2019 for beneficiaries ages 0 to 18 years old to measure PSG utilization based on the child’s residential state and ZIP code. We also examine the correlation between PSG rates and race/ethnicity and rurality. Medicaid/CHIP is the largest payer of insurance for children in the USA, and the only source of national data on pediatric healthcare utilization. The results of this Medicaid population-based study can provide valuable insight into utilization of PSG and its variation across locations and geographic characteristics.

## Methods

### Study data source

This study used the Transformed Medicaid Statistical Information System (T-MSIS) Analytic Files (TAF) to select Medicaid/CHIP enrollees ages 0 to 18 years in 2017 to 2019 from 49 states and the District of Columbia [10]. Rhode Island was excluded due to missing ZIP code for all years of the study as well as being an outlier for low number of PSG claims. We did not include data for 2020 and later because of the potential for the pandemic-related changes in healthcare utilization and Medicaid enrollment to affect the reliability and generalizability of the utilization estimates. In particular, the federal Medicaid continuous enrollment requirement likely kept children enrolled even after they moved out of state or gained employer-based coverage and were no longer using Medicaid services. This created an artificial inflation of Medicaid enrollment because individuals and families were not disenrolled when their eligibility effectively ended [11].

Person-years were aggregated from monthly age-specific enrollment for all Medicaid/CHIP enrollees who did not have restricted benefits, were not dual-eligible, had a known date of birth, and had at least one inpatient or outpatient service utilization claim in the specific enrollment year. We used outpatient claims to identify overnight attended PSG (CPT codes 95,782, 95,783, 95,807, 95,808, 95,810, and 95,811) among beneficiaries who met these criteria. Age on the last day of the month was used for all beneficiaries to assign age for analysis.

### Geography

We conducted geographic analysis at the US Census Division, state and local levels. Local areas were based on the beneficiary’s last-in-year ZIP code as reported by the state Medicaid programs. We used the US Postal Service PostalPro ZIP Locale Detail file to validate ZIP codes and convert all delivery ZIP codes to physical (parent) ZIP codes [12]. These validated and converted ZIP codes were then aggregated to approximate the US Census Bureau Public Use Microdata Areas (PUMAs) [13]. PUMAs are contiguous geographic areas with a minimum of 100,000 residents, ranging from neighborhoods within cities to multiple counties in rural areas. Compared to administratively defined local areas such as ZIP codes and counties, PUMAs reduce heteroscedasticity in mapping and exploration of geographic variation. Although the aggregated ZIP codes do not precisely match the boundaries of the census-tract based PUMA maps, applying the same ZIP code definition to the Medicaid data ensured correspondence between the numerator and denominator. Since PUMAs are too large to be useful to measure neighborhood characteristics, we used ZIP code level data to examine the correlations between PSG rates and race/ethnicity and urban/rural settings. Beneficiary address information other than ZIP code was not available. For analysis based on ZIP codes or PUMAs, beneficiaries with missing or invalid ZIP codes were excluded. Vermont, which was missing ZIP code for 25% of children, was also excluded from the ZIP code or PUMA-based analyses.

Urbanicity was based on the 2010 Rural/Urban Commuting Area (RUCA) codes for ZIP codes, defined as metropolitan, micropolitan, small rural towns, and isolated rural towns [14]. Race and ethnicity distribution of ZIP codes was obtained from the American Community Survey 5-year ZIP Code Tabulation Area (ZCTA) estimates for 2015–2019 [15]. We used a one-to-one match between ZCTA and ZIP code, which resulted in some ZIP codes being excluded. Six groupings of race and ethnicity distribution types were made to represent the majority race/ethnicity population of the ZIP code. ZIP codes were assigned to groups if their population was 50% or more Black non-Hispanic, Hispanic, White non-Hispanic, or other race/ethnicity, which included Asian, Hawaiian and other Pacific Islander, and American Indian/Alaskan Native. Since over 85% of the total ZIP codes were 50% or more White non-Hispanic, this group was further divided into those that were 50–89% White non-Hispanic (46%) and those that were 90% or more White non-Hispanic (53%). The sixth group was all ZIP codes for which there was no majority race/ethnicity group. We were unable to use individual level self-reported race and ethnicity, because the majority of states had high levels of missing data or misclassification [16].

### Statistical analysis

For visualization and for examining variation, age-adjusted rates of PSG were calculated for states and PUMAs, standardized to the distribution of eligible person-years included in the study. Jenks natural breaks optimization method was used for determining groupings for the mapped rate data. The Jenks method is a goodness of variance fit technique that optimizes between-group variances [17]. PUMAs with 11 or fewer cases were considered to have unstable rates and were excluded from map of rates by PUMAs. Differences in the distribution of PUMA rates by US Census Division were assessed for statistical significance using the Games–Howell test for differences of means, which adjusts for multiple comparisons and does not require homogeneity of variances [18]. Variability of rates by state and PUMAs was measured using the interquartile range and the coefficient of variation (CoV), defined as the standard deviation divided by the mean.

For examining the association between PSG rates and demographics, multivariable Poisson regression with clustered error variance estimators at the ZIP code level was used to derive rates and rate differences with 95% confidence intervals (CI). These models included beneficiary age, ZIP code level race/ethnicity majority group, and ZIP code level urban/rural category.

Due to the varying quality and completeness of Medicaid data reported from states, we conducted a sensitivity analysis of the ZIP code level regression excluding states with (1) overall completeness concerns, (2) concerns over the completeness and quality of procedure codes, and (3) either overall completeness or procedure related concerns (Supplemental Figure S2). According to the Medicaid Data Quality Atlas, there were 14 states that were categorized as having “unusable” data for number of enrollees or claim volume for at least 1 year from 2017 to 2019 (Arkansas, Colorado, Florida, Idaho, Maine, Montana, New Mexico, New York, North Dakota, Pennsylvania, Tennessee, Mississippi, Missouri, and Nebraska) [19]. Six states had high percent of procedure codes that were missing or invalid on outpatient claims (Georgia, New York, Pennsylvania, Texas, Utah, and Illinois) [19].

All analyses were conducted in SAS Enterprise Guide v8.5, SAS Institute Inc., Cary NC. Maps and box plot were created in SAS. This study was approved by the Nationwide Children’s Hospital Institutional Review Board.

## Results

There were 51,724,769 children aged 0 to 18 years enrolled with unrestricted benefits for at least 1 month between 2017 and 2019. Beneficiaries were excluded for missing birth date (129, 0.0%), lack of inpatient or outpatient service use claim (5,477,869, 11%), or enrollment in Rhode Island (131,776, 0.3%) (Online Resource Figure S1). The remaining 46,114,998 beneficiaries each contributed an average of 2 person-years of observation to the study (95,478,712 person-years). Among beneficiaries ages 1 to 17, most person-years (92.0%) were associated with 11 or 12 months of enrollment each year, and only 1.2% of person-years were associated with beneficiaries enrolled for fewer than 6 months. Excluding Vermont, there were 538,431 (1.2%) children with missing ZIP code information, representing 0.8% of person-years of observation (see Online Resource Table S1 for ZIP code missingness by state). Of the 23,898 valid ZIP codes in the study, 1571 (6.8% of ZIP codes, 3.9% of person-years) did not match Census ZCTAs, resulting in 90,293,931 (81.8%) person-years being included in the regression analysis.

We identified 482,688 PSGs in the claims data for 0–18-year-olds from 2017 to 2019. Of these, 1853 (0.4%) were excluded due to restricted benefits or dual eligibility. Polysomnograms performed among beneficiaries from Rhode Island (849, 0.2%) were excluded from all analyses, and those from Vermont (685, 0.1%) or who were missing ZIP code (2719, 0.6%) were excluded from PUMA analyses. A further 21,013 (4.4%) were not included in the regression analysis because the ZIP code did not match to a Census ZCTA record. These exclusions resulted in 478,568 (99.1%) PSGs included in the state level analysis, 474,315 (98.3%) included in the PUMA analysis, and 453,302 (93.9%) included in the ZIP code level regression (Online Resource Figure S1).

### Geographic patterns

**Fig. 1:**
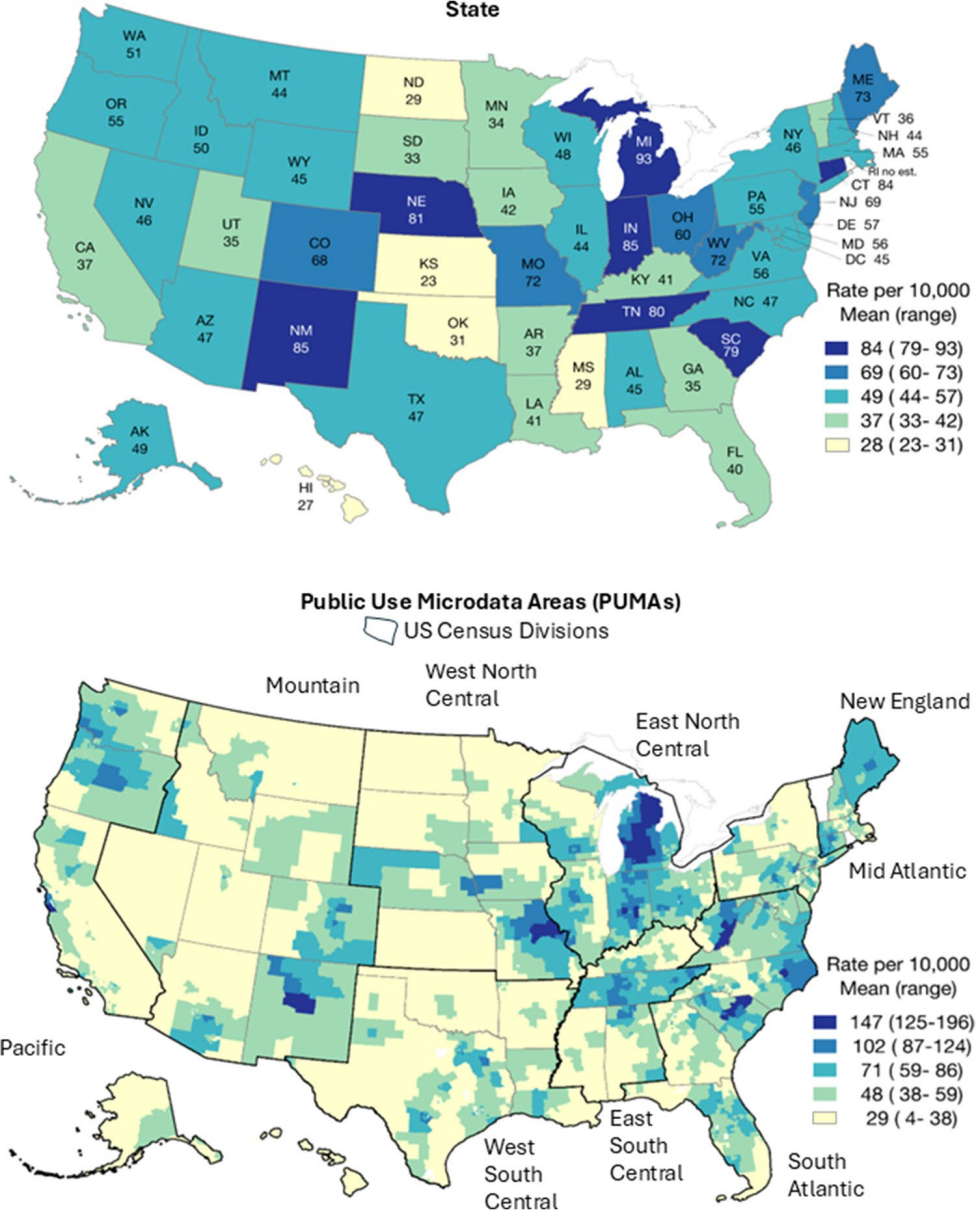
Age-adjusted polysomnography rates per 10,000 Medicaid beneficiaries ages 0–18 years, 2017–2019

There were 50.1 PSGs per 10,000 enrolled Medicaid person-years. There was a fourfold difference in age-adjusted PSG rates at the state level, from 23 cases per 10,000 person-years in Kansas to 93 cases per 10,000 person-years in Michigan (Fig. 1). Distributions of rates within states were heterogeneous, with some states having uniform rates at the PUMA level (e.g., Kansas, Maine, and Michigan) while other states had focal points with a wide range of rates (e.g., Missouri, California, and South Carolina) (Fig. 1).

**Fig. 2:**
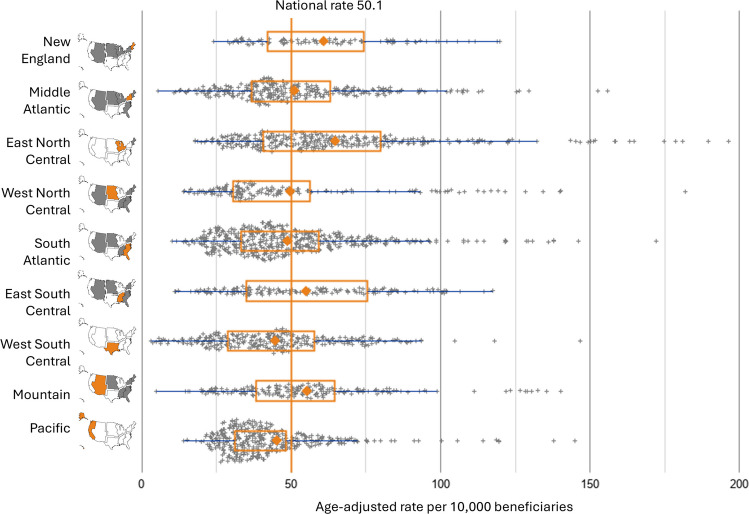
US Census Division distribution of Public Use Microdata Area (PUMA) polysomnography rates per 10,000 person-years, Medicaid beneficiaries ages 0–18 years, 2017–2019. + = one Public Use Microdata Area. Divisions shown in gray on maps are not statistically significantly different at the 0.05 level

The largest concentration of PUMAs with higher rates was in the East North Central Census Division, which had the highest mean PUMA rates (64.9 per 10,000) as well as a wide range of rates (Fig. 2, Table 1). Of the 25 PUMAs in the highest 1% of rates (age-adjusted rates greater than 140 per 10,000), 16 were in the East North Central Division, with 15 in Michigan. The Pacific (39.6) and West South Central (43.7) Divisions had the lowest rates, and their PUMA distributions were statistically different from each other and all other divisions (Table 2). Among the 25 PUMAs with the lowest rates (less than 13 cases per 10,000), 14 were in Texas. The mean PUMA rates for the Middle Atlantic, South Atlantic, West North Central Divisions were close to the national rate, although the West North Central Division has the highest CoV of all regions. This can be seen visually in Fig. 1, where we see a broad band of high to moderate rates across Missouri, Iowa, and Nebraska, surrounded by low rates in Kansas to the south and Minnesota and the Dakotas to the north.

**Table 1:** Characteristics of US Census Region and Division distribution of Public Use Microdata Area (PUMAs) polysomnography rates per 10,000 person-years, Medicaid beneficiaries ages 0–18 years, 2017–2019

				Characteristics of polysomnograms by PUMAs within locality						
	Polysomnograms^a^	Person-years^a^	Age-adjusted rate	Number of PUMAs	Mean rate	Median rate	Interquartile range	Minimum rate	Maximum rate	Coefficient of variation
USA	477,719	95,478,212	50.1	2,407	51.8	45.5	29.2	3.1	1157.9	66.2
Northeast	83,518	15,334,984	54.8	409	53.5	47.5	30.1	5.2	156.0	45.1
New England^b^	21,615	3,488,576	62.4	98	61.0	58.5	32.2	24.0	119.8	37.7
Middle Atlantic	61,903	11,846,408	52.5	311	51.1	45.4	26.4	5.2	156.0	46.9
Midwest	108,886	18,257,993	59.7	503	59.9	52.2	40.3	14.0	196.4	55.1
East North Central	82,489	12,836,693	64.4	338	64.9	57.5	39.3	17.7	196.4	50.8
West North Central	26,397	5,421,299	48.7	165	49.6	37.6	25.8	14.0	182.1	61.9
South	178,883	37,695,069	47.6	934	48.6	44.8	29.0	3.1	172.2	48.1
South Atlantic	84,196	17,230,050	49.0	488	48.9	44.1	25.8	10.0	172.2	47.6
East South Central	32,972	6,321,383	52.3	153	55.1	51.6	40.3	11.0	117.3	45.7
West South Central	61,715	14,143,636	43.7	293	44.8	44.0	29.0	3.1	146.8	48.8
West	106,432	24,190,166	44.0	561	48.5	41.5	23.5	4.8	1157.9	106.2
Mountain	38,410	7,023,196	54.8	184	55.2	51.9	26.6	4.8	140.1	45.7
Pacific	68,022	17,166,970	39.6	377	45.3	38.4	17.0	14.0	1157.9	132.8

^a^Includes only polysomnograms and person-years among children with known ZIP code

^b^New England excludes Rhode Island and Vermont due to high levels of missing ZIP codes

**Table 2:** US Census Division pairwise comparisons of statistical significance of differences in mean polysomnography Public Use Microdata Area (PUMAs) rates per 10,000 person-years, Medicaid beneficiaries ages 0–18 years, 2017–2019

	New England^a^	Middle Atlantic	East North Central	West North Central	South Atlantic	East South Central	West South Central	Mountain	Pacific
New England	1								
Middle Atlantic	0.1392	1							
East North Central	0.3110	< 0.0001^*^	1						
West North Central	0.8395	0.9991	0.0064^*^	1					
South Atlantic	0.0011^*^	0.5654	< 0.0001^*^	0.5612	1				
East South Central	0.0380^*^	0.9976	< 0.0001^*^	0.9557	0.9927	1			
West South Central	< 0.0001^*^	< 0.0001^*^	< 0.0001^*^	0.0002^*^	< 0.0001^*^	< 0.0001^*^	1		
Mountain	0.5956	0.9994	0.0004^*^	1.0	0.3495	0.9380	< 0.0001^*^	1	
Pacific	< 0.0001^*^	< 0.0001^*^	< 0.0001^*^	< 0.0001^*^	< 0.0001^*^	< 0.0001^*^	0.0029^*^	< 0.0001^*^	1

^*^Statistically significant at the 0.05 level

^a^New England excludes Rhode Island and Vermont due to high levels of missing ZIP codes

### Urbanicity and racial/ethnic geographic characteristics

Both urbanicity and higher percent White race were associated with higher rates of PSG at the ZIP code level (Table 3). The metropolitan ZIP code group, which accounts for 84.1% of the PSGs in the study, had the highest adjusted mean rate (41.3 per 10,000), with rates decreasing as degree of rurality increased. The race-ethnicity majority category that included ZIP codes with ≥ 90% White residents accounted for 12% of PSGs and had the highest rate at 50.8 per 10,000, with the lowest PSG rate seen in ZIP codes with more than 50% “other” races (27.6 per 10,000). While the model-based rate differences were statistically significant, some differences were small. Compared to metropolitan ZIP codes, the more rural ZIP code categories ranged from 6.0 to 9.7 per 10,000 fewer PSGs. For race-ethnicity, ZIP codes with higher proportion people of color ranged from 4.2 to 23.2 per 10,000 fewer PSGs compared to ZIP codes with 90% or more White population.

**Table 3:** Association between polysomnography and ZIP code level race-ethnicity and rurality characteristics, Medicaid beneficiaries ages 0–18 years, 2017–2019

	Polysomnography		Person-years		ZIP codes		Rate per 10,000^a^	95% confidence interval		Rate difference per 10,000^a^	95% confidence interval		*p* value for rate difference
	*n*	%	*n*	%	*n*	%							
Rural–Urban Commuting Area classification													
Metropolitan	381,295	84.1	74,183,823	82.2	11,771	52.7	47.3	46.0	48.6	0	[Reference]		
Micropolitan	41,158	9.1	8,962,138	9.9	3351	15.0	41.3	39.5	43.1	− 6.0	− 7.8	− 4.2	< 0.0001
Small rural town	19,692	4.3	4,540,291	5.0	2624	11.8	38.4	36.8	40.1	− 8.9	− 11	− 7.2	< 0.0001
Isolated rural town	11,157	2.5	2,607,678	2.9	4581	20.5	37.6	35.8	39.4	− 9.7	− 12	− 7.9	< 0.0001
Race-ethnicity majority													
No majority	76,217	16.8	15,776,962	17.5	1100	4.9	41.1	39.1	43.2	− 9.7	− 12.0	− 7.3	< 0.0001
≥ 50% Black	46,899	10.4	8,592,514	9.5	840	3.8	46.5	44.3	48.9	− 4.2	− 6.8	− 1.7	< 0.0001
≥ 50% Hispanic	71,547	15.8	15,330,443	17.0	908	4.1	39.8	37.5	42.1	− 11.0	− 13.6	− 8.4	0.0013
≥ 50% other races	3548	0.8	1,170,175	1.3	429	1.9	27.6	24.5	31.0	− 23.2	− 26.7	− 19.7	< 0.0001
≥ 50– < 90% White	198,750	43.8	39,073,611	43.3	8810	39.5	44.4	43.3	45.5	− 6.3	− 8.0	− 4.7	< 0.0001
≥ 90% White	56,341	12.4	10,350,226	11.5	10,240	45.9	50.8	49.4	52.1	0	[Reference]		

^a^Rate and rate difference from Poisson regression with clustered error variance estimators at the ZIP code level, adjusted for age, ZIP code race-ethnicity majority population and Rural–Urban Commuting Area classification

Restricting the data to states with higher quality data in the sensitivity analysis resulted in minimal changes in the results (Online Resource Table S2). Mean rates for all ZIP code groupings were within 10% of the means derived using all states. The mean PSG rates for ZIP codes with 50% or higher Hispanic population were the most affected by these restrictions, with means that were 8 to 10% lower than when all states were included. Several states with a higher proportion of Hispanic residents overall (Texas, New Mexico, and Florida) were excluded in this sensitivity analysis due to data concerns (Online Resource Figure S2). The rate differences were in the same direction and magnitude compared to the analysis of all states.

## Discussion

To our knowledge, this is the first population-based study to examine the geography and use of PSG in US children. We specifically examined PSG among children enrolled in Medicaid, which is the only data source for children with national coverage and residence location information and is also the largest payer for child health care in the USA. We found that PSG use in the Medicaid pediatric population has a high degree of variation across the USA, with fourfold difference in rates by state. The lowest rates were observed in the Pacific (e.g., California) and West South Central (Oklahoma) Census Divisions, and the highest were in the East North Central (Michigan) and New England (Connecticut). It is worth noting that low utilization of PSG in California children with Medicaid mirrors prior data on limited access to care and tonsillectomy utilization for children with Medicaid in this region [20, 21]. ZIP codes in metropolitan settings and those with 90% or greater White non-Hispanic residents had the highest PSG rates. The rate differences, however, were small. For example, the difference in rates between metropolitan settings and isolated rural settings was less than 0.1%.

Like many healthcare tests, PSG falls into a “gray zone” of appropriate use, with a balance between benefits and limitations for many children [22]. This balance varies by the indication for PSG but is of particular concern with respect to oSDB. Professional groups in the USA differ in recommendations for PSG referral for children with habitual snoring and other signs and symptoms of OSA [23]. Guidelines from the American Academy of Sleep Medicine (AASM, 2011) and the American Academy of Pediatrics (AAP, 2012) support PSG for all children with suspected OSA [2, 4]. The more recent American Academy of Otolaryngology–Head and Neck Surgery (AAO-HNS, 2019) guidelines take a more limited approach in recommending PSG when there are elevated anesthesia or surgical risks (e.g., obesity, Down syndrome) or when the need for tonsillectomy is not clear [24]. This recommendation is based on the known benefits of treating oSDB in relation to both the safety of tonsillectomy and the expense and difficulty in obtaining PSG for many families [24–26].

It is likely that clinician experience with use of PSG as well as knowledge of local pediatric PSG availability and wait times influences their likelihood of providing referrals to individual patients, insofar as bypassing PSG based on availability has been incorporated into the AAP guideline [4]. Likewise, qualitative research on caregiver preference points to barriers to PSG [27], which could contribute to observed geographic variation in utilization. Referral to PSG for suspected oSDB has been shown to increase loss-to-follow-up, time to surgery, and cost [28–30]. Recent studies have also suggested that PSG may contribute to racial and ethnic disparity in tonsillectomy in the Medicaid population [31–34]. Another pragmatic concern for use of PSG among children with oSDB prior to tonsillectomy is that it could limit availability of pediatric PSG for other indications [35].

Radhakrishnan et al. observed that shorter travel times increased the likelihood of PSG for obstructive sleep breathing problems among children in Ontario, Canada [36]. Longer travel time to pediatric sleep clinics is a possible explanation for our finding of lower PSG rates in rural settings. Likewise, transportation and other deprivation-based barriers may have contributed to the lower rates we observed among ZIP codes with higher percentages of non-White residents [37]. Like all claims-based analyses, our study was limited in the type of data available. Lack of data on the locations of sleep labs with pediatric capacity as well as poor quality provider location data on the Medicaid claims meant that assessing access measures, such as travel distance to PSG, was not feasible. While detailed data on the geographic distribution of the underlying prevalence of pediatric sleep disorders is likewise lacking, regional differences have been observed. The highest prevalence of self-reported short sleep duration among both children and adults has been observed in the southeast and central Appalachia while the lowest prevalence is in the Northwest, Mountain, and Upper Midwest regions [38–40].

Another important limitation for the use of Medicaid data for national-level analysis is the varying quality and completeness of data reported by state Medicaid programs. Unlike Medicare data collection systems, the T-MSIS system was designed to collect both fee-for-service and managed care claims. There are concerns about the quality and completeness of claims reporting by managed care, although the number of states with such concerns has decreased in the years covered by this study [41]. Missing or invalid procedure codes, particularly those reported from facilities, were also a concern for several states [19]. A sensitivity analysis (Online Resource Table S2 and Figure S2) for the associations between PSG and urban/rural and race-ethnicity at the ZIP code level showed that the interpretation of the results did not change when states with greatest reporting concerns were excluded.

Medicaid is the largest healthcare payer in the USA for children, representing approximately 50% of all insured children. Medicaid coverage, however, varies geographically based on state eligibility policies. Coverage also varies by urban–rural setting and race/ethnicity in relation to the patterns of poverty in the USA, which also contributes to differential coverage by state [42]. The estimates presented here, therefore, cannot be used at the local level to plan either individual care or changes in service provision patterns.

## Conclusion

The data on PSG utilization in Medicaid presented here can inform efforts to reduce variation in evaluation and treatment for children with oSDB, provide wider and more facile access to PSG, and modify practice guidelines for referral to PSG. Given the risks of delay in care and loss-to-follow-up as well as the benefit of PSG testing in guiding treatment recommendations across pediatric sleep disorders, understanding the varying levels of practice patterns, access, and utilization of PSG among children across the country requires further research in availability and barriers in terms of both geography and other dimensions of access such as diversity, affluence, and the structural factors of healthcare.

## Supplementary Information

Below is the link to the electronic supplementary material.

ESM 1 (DOCX 265 KB)

## Data Availability

Data provided by the Centers for Medicare and Medicaid Services under Data Use Agreement RSCH-2022–58522. Data cannot be shared.
