# Supplementary material for: Geographic and racial/ethnic patterns of polysomnography use among children enrolled in Medicaid, 2017–2019
Source: J Clin Sleep Med. 2026 Aug 3;22(1):128. doi: 10.1007/s44470-026-00149-w (PMC13433656; doi:10.1007/s44470-026-00149-w)
Supplement: Supplementary file 1 [file 44470_2026_149_MOESM1_ESM.docx]

Journal of Clinical Sleep Medicine

Online Resource

Geographic and racial/ethnic patterns of polysomnography use among children enrolled in Medicaid, 2017-2019

Colleen C. McLaughlin PhD^a^, Jesse L. Hawke PhD^a^, Emily F. Boss MPH MD^b^, Guy M. Brock PhD^c^, Meredith M. Lind MD^d^, Tasleem J. Padamsee PhD^e^, Prasanth Pattisapu MPH MD^d^, Deena J. Chisolm PhD^a,f^, Laura J. Chavez PhD^a,f^

a. Center for Child Health Equity and Outcomes Research, Abigail Wexner Research Institute at Nationwide Children’s Hospital, 700 Children’s Drive, Columbus, OH, USA
b. Department of Otolaryngology, Johns Hopkins University School of Medicine, 601 N. Caroline Street, Baltimore, MD, USA
c. Department of Biomedical Informatics and Center for Biostatistics, Ohio State University, 250 Cunz Hall, 1841 Neil Ave, Columbus, OH, USA
d. Department of Otolaryngology, Ohio State University and Nationwide Children's Hospital, 700 Children’s Drive, Columbus, OH, USA
e. Division of Health Services Management and Policy, College of Public Health, Ohio State University, 280F Cunz Hall, 1841 Neil Ave, Columbus, OH, USA
f. Department of Pediatrics, College of Medicine, Ohio State University, 700 Children’s Drive, Columbus, OH

Corresponding author:

Colleen McLaughlin, MPH, PhD

Abigail Wexner Research Institute at Nationwide Children’s Hospital

700 Children’s Drive, Columbus, OH, 43205

[colleen.mclaughlin@nationwidechildrens.org](mailto:colleen.mclaughlin@nationwidechildrens.org)

(518) 505-6868 (cell)

orcid.org/0000-0003-4172-8172

Jesse.Hawke@nationwidechildrens.org

[Boss@jhu.edu](mailto:Boss@jhu.edu) (Emily Boss)

Guy.Brock@osumc.edu

Meredith.Lind@nationwidechildrens.org

[padamsee.1@osu.edu](mailto:padamsee.1@osu.edu) (Tasleem Padamsee)

Prasanth.Pattisapu@nationwidechildrens.org

Deena.Chisolm@nationwidechildrens.org

Laura.Chavez@nationwidechildrens.org

**Fig. S1 Study Subject Selection**

**Table S1 Percent of person-years included in state level analysis that are not included in ZIP Code and PUMAs analyses, by state**

| State^a^ | Person-years^b^ | Missing ZIP Code and PUMA (%) |  | State^a^ | Person-years^b^ | Missing ZIP Code and PUMA (%) |
| --- | --- | --- | --- | --- | --- | --- |
| Alabama | 1,613,419 | 0.08 |  | Mississippi | 1,346,100 | 0.07 |
| Alaska | 268,392 | 1.11 |  | Missouri | 1,743,676 | 0.67 |
| Arizona | 2,501,081 | 2.80 |  | Montana | 381,728 | 0.16 |
| Arkansas | 1,349,630 | 2.94 |  | Nebraska | 510,664 | 0.14 |
| California | 14,734,268 | 1.73 |  | Nevada | 899,344 | 0.06 |
| Colorado | 1,652,802 | 0.04 |  | New Hampshire | 280,465 | 0.30 |
| Connecticut | 1,071,571 | 0.23 |  | New Jersey | 2,342,492 | 0.03 |
| Delaware | 329,757 | 0.04 |  | New Mexico | 1,031,420 | 0.74 |
| District of Columbia | 245,489 | 0.04 |  | New York | 7,417,849 | 7.62 |
| Florida | 4,330,123 | 0.11 |  | North Carolina | 3,668,308 | 0.25 |
| Georgia | 3,979,547 | 0.01 |  | North Dakota | 137,960 | 0.32 |
| Hawaii | 422,861 | 0.35 |  | Ohio | 3,628,388 | 0.13 |
| Idaho | 616,455 | 0.14 |  | Oklahoma | 1,540,019 | 0.04 |
| Illinois | 4,112,440 | 0.01 |  | Oregon | 1,255,302 | 1.89 |
| Indiana | 1,983,246 | 0.04 |  | Pennsylvania | 3,743,398 | 0.43 |
| Iowa | 1,035,239 | 1.18 |  | South Carolina | 1,874,121 | 0.52 |
| Kansas | 857,371 | 0.06 |  | South Dakota | 243,098 | 0.03 |
| Kentucky | 1,732,767 | 0.33 |  | Tennessee | 2,233,096 | 0.16 |
| Louisiana | 2,263,731 | 0.05 |  | Texas | 10,792,346 | 0.14 |
| Maine | 329,879 | 0.88 |  | Utah | 622,269 | 0.04 |
| Maryland | 1,893,680 | 0.01 |  | Virginia | 1,903,617 | 0.30 |
| Massachusetts | 1,954,693 | 0.08 |  | Washington | 2,405,605 | 0.02 |
| Michigan | 2,925,841 | 0.01 |  | West Virginia | 622,356 | 0.28 |
| Minnesota | 1,544,536 | 0.02 |  | Wisconsin | 1,512,042 | 0.03 |
|  |  |  |  | Wyoming | 123,954 | 0.45 |

a. RI and VT excluded from ZIP Code level analysis due to high percentage missingness
b. Beneficiary count not included because the number of cases missing ZIP Code falls below the threshold for reporting (<12 cases) for many states

**Sensitivity analysis methods and results**

Due to the varying quality and completeness of Medicaid data reported from states, we conducted a sensitivity analysis of the ZIP Code level regression excluding states with 1) overall completeness concerns (n=14), 2) concerns over the completeness and quality of procedure codes (n=6), and 3) either overall completeness or procedure related concerns (n=18) (details provided in Supplemental Table x and Figure x). According to the Medicaid Data Quality Atlas, there were 14 states that were categorized as having “unusable” data for number of enrollees or claim volume for at least one year from 2017 to 2019 (Arkansas, Colorado, Florida, Idaho, Maine, Montana, New Mexico, New York, North Dakota, Mississippi, Missouri, Nebraska, Pennsylvania, Tennessee).^1^ Six states had high percent of procedure codes that were missing or invalid on outpatient claims (Georgia, Illinois, New York, Pennsylvania, Texas, Utah).^1^

Restricting the data to states with higher quality data in the sensitivity analysis resulted in minimal changes in the results (Supplemental Table S2). Mean rates for all ZIP Code groupings were within 10% of the means derived using all states. The mean PSG rates for ZIP Codes with 50% or higher Hispanic population were the most affected by these restrictions, with means that were 8% to 10% lower than when all states were included. Several states with higher proportion of Hispanic residents overall (Texas, New Mexico, Florida) were excluded in this sensitivity analysis due to data concerns (Supplemental Figure S2). The rate differences were in the same direction and magnitude compared to the analysis of all states.

1.	Centers for Medicare and Medicaid Services. DQ Atlas. Accessed 8/10/2023, https://www.medicaid.gov/dq-atlas

**Table S2 Sensitivity analysis results: using alternate groupings of states based on data quality to examine the association between polysomnography and ZIP Code level race-ethnicity and rurality characteristics, Medicaid beneficiaries ages 0-18 years, 2017-2019**

|  | Polysomnograms | | Person-years | | ZIP Codes | | Rate per 10,000 | | | Rate difference per 10,000 | | | |
| --- | --- | --- | --- | --- | --- | --- | --- | --- | --- | --- | --- | --- | --- |
|  | n | % | n | % | n | % | Rate | 95% CI | | Rate | 95% CI | | p-value |
| **All States** |  |  |  |  |  |  |  |  |  |  |  |  |  |
| Rural-Urban Commuting Area Classification | | | | | | | | | | | | | |
| Metropolitan | 381,295 | 84.1 | 74,183,823 | 82.2 | 11,771 | 52.7 | 41.3 | 39.5 | 43.1 | 0.0 | [reference] | |  |
| Micropolitan | 41,158 | 9.1 | 8,962,138 | 9.9 | 3,351 | 15.0 | 38.4 | 36.8 | 40.1 | -6.0 | -7.8 | -4.2 | <.0001 |
| Small rural town | 19,692 | 4.3 | 4,540,291 | 5.0 | 2,624 | 11.8 | 37.6 | 35.8 | 39.4 | -8.9 | -11.0 | -7.2 | <.0001 |
| Isolated rural town | 11,157 | 2.5 | 2,607,678 | 2.9 | 4,581 | 20.5 | 47.3 | 46.0 | 48.6 | -9.7 | -12.0 | -7.9 | <.0001 |
| Race-ethnicity majority | | | | | | | | | | | | | |
| No majority | 76,217 | 16.8 | 15,776,962 | 17.5 | 1,100 | 4.9 | 41.1 | 39.1 | 43.2 | -9.7 | -12.0 | -7.3 | <.0001 |
| >=50% Black | 46,899 | 10.4 | 8,592,514 | 9.5 | 840 | 3.8 | 46.5 | 44.3 | 48.9 | -4.2 | -6.8 | -1.7 | <.0001 |
| >=50% Hispanic | 71,547 | 15.8 | 15,330,443 | 17.0 | 908 | 4.1 | 39.8 | 37.5 | 42.1 | -11.0 | -13.6 | -8.4 | 0.0013 |
| >=50% Other races | 3,548 | 0.8 | 1,170,175 | 1.3 | 429 | 1.9 | 27.6 | 24.5 | 31.0 | -23.2 | -26.7 | -19.7 | <.0001 |
| >=50%-<90% White | 198,750 | 43.8 | 39,073,611 | 43.3 | 8,810 | 39.5 | 44.4 | 43.3 | 45.5 | -6.3 | -8.0 | -4.7 | <.0001 |
| >=90% White | 56,341 | 12.4 | 10,350,226 | 11.5 | 10,240 | 45.9 | 50.8 | 49.4 | 52.1 | 0.0 | [reference] | |  |
| **Group 1: Usable Completeness Data^a^** | | | | | | | | | | | | | |
| Rural-Urban Commuting Area Classification | | | | | | | | | | | | | |
| Metropolitan | 280,146 | 84.4 | 56,040,557 | 82.9 | 8,166 | 53.6 | 46.1 | 44.9 | 47.4 | 0.0 | [reference] | |  |
| Micropolitan | 30,232 | 9.1 | 6,574,856 | 9.7 | 2,274 | 14.9 | 41.1 | 39.1 | 43.2 | -5.1 | -7.2 | -3.0 | <.0001 |
| Small rural town | 13,837 | 4.2 | 3,204,157 | 4.7 | 1,818 | 11.9 | 37.6 | 35.7 | 39.5 | -8.6 | -10.5 | -6.6 | <.0001 |
| Isolated rural town | 7,895 | 2.4 | 1,800,416 | 2.7 | 2,991 | 19.6 | 37.5 | 35.4 | 39.7 | -8.6 | -10.9 | -6.4 | <.0001 |
| Race-ethnicity majority | | | | | | | | | | | | | |
| No majority | 56,411 | 17.0 | 12,203,699 | 18.0 | 837 | 5.5 | 39.8 | 37.8 | 41.9 | -12.6 | -15.2 | -10.0 | <.0001 |
| >=50% Black | 32,497 | 9.8 | 6,009,811 | 8.9 | 560 | 3.7 | 46.5 | 43.8 | 49.4 | -5.9 | -9.1 | -2.7 | 0.0003 |
| >=50% Hispanic | 57,407 | 17.3 | 13,027,691 | 19.3 | 675 | 4.4 | 37.9 | 35.7 | 40.3 | -14.5 | -17.3 | -11.6 | <.0001 |
| >=50% Other races | 2,322 | 0.7 | 815,029 | 1.2 | 346 | 2.3 | 26.6 | 23.8 | 29.8 | -25.7 | -29.2 | -22.3 | <.0001 |
| >=50%-<90% White | 145,939 | 43.9 | 28,832,246 | 42.6 | 6,382 | 41.9 | 44.7 | 43.4 | 46.0 | -7.7 | -9.8 | -5.6 | <.0001 |
| >=90% White | 37,534 | 11.3 | 6,731,509 | 10.0 | 6,449 | 42.3 | 52.4 | 50.6 | 54.2 | 0.0 | [reference] | | |
| **Group 2: Usable Outpatient Procedure Data^b^** | | | | | | | | | | | | | |
| Rural-Urban Commuting Area Classification | | | | | | | | | | | | | |
| Metropolitan | 275,723 | 81.9 | 51,431,000 | 79.7 | 8,246 | 49.5 | 48.7 | 47.4 | 50.1 | 0.0 | [reference] | | |
| Micropolitan | 34,403 | 10.2 | 7,196,200 | 11.2 | 2,555 | 15.3 | 42.0 | 40.0 | 44.0 | -6.7 | -8.8 | -4.7 | <.0001 |
| Small rural town | 16,666 | 5.0 | 3,655,968 | 5.7 | 1,988 | 11.9 | 39.3 | 37.5 | 41.2 | -9.4 | -11.3 | -7.5 | <.0001 |
| Isolated rural town | 9,829 | 2.9 | 2,218,161 | 3.4 | 3,859 | 23.2 | 37.9 | 36.0 | 39.9 | -10.8 | -12.8 | -8.8 | <.0001 |
| Race-ethnicity majority | | | | | | | | | | | | | |
| No majority | 49,051 | 14.6 | 10,016,607 | 15.5 | 749 | 4.5 | 41.4 | 39.1 | 43.9 | -12.3 | -15.1 | -9.6 | <.0001 |
| >=50% Black | 32,421 | 9.6 | 5,637,047 | 8.7 | 660 | 4.0 | 48.9 | 46.0 | 51.9 | -4.8 | -8.1 | -1.6 | 0.0033 |
| >=50% Hispanic | 44,102 | 13.1 | 9,748,258 | 15.1 | 570 | 3.4 | 38.1 | 35.3 | 41.1 | -15.7 | -18.9 | -12.4 | <.0001 |
| >=50% Other races | 2,753 | 0.8 | 932,842 | 1.4 | 413 | 2.5 | 27.5 | 24.8 | 30.6 | -26.2 | -29.5 | -22.9 | <.0001 |
| >=50%-<90% White | 161,054 | 47.8 | 29,961,315 | 46.5 | 6,651 | 40.0 | 46.7 | 45.4 | 48.0 | -7.0 | -9.0 | -5.1 | <.0001 |
| >=90% White | 47,240 | 14.0 | 8,205,260 | 12.7 | 7,605 | 45.7 | 53.7 | 52.2 | 55.3 | 0.0 | [reference] | | |
| **Group 3: Usable Completeness and Procedure Data^c^** | | | | | | | | | | | | | |
| Rural-Urban Commuting Area Classification | | | | | | | | | | | | | |
| Metropolitan | 214,814 | 83.1 | 41,315,510 | 81.5 | 6,394 | 52.4 | 47.3 | 45.8 | 48.7 | 0.0 | [reference] | | |
| Micropolitan | 25,371 | 9.8 | 5,292,816 | 10.4 | 1,836 | 15.0 | 42.1 | 39.8 | 44.5 | -5.2 | -7.6 | -2.8 | <.0001 |
| Small rural town | 11,408 | 4.4 | 2,516,680 | 5.0 | 1,365 | 11.2 | 39.1 | 36.9 | 41.4 | -8.2 | -10.5 | -5.8 | <.0001 |
| Isolated rural town | 7,049 | 2.7 | 1,566,920 | 3.1 | 2,606 | 21.4 | 38.4 | 36.1 | 40.9 | -8.9 | -11.4 | -6.4 | <.0001 |
| Race-ethnicity majority | | | | | | | | | | | | | |
| No majority | 39,335 | 15.2 | 8,288,699 | 16.4 | 589 | 4.8 | 41.0 | 38.5 | 43.6 | -12.2 | -15.2 | -9.1 | <.0001 |
| >=50% Black | 24,106 | 9.3 | 4,094,759 | 8.1 | 426 | 3.5 | 50.8 | 47.3 | 54.6 | -2.3 | -6.3 | 1.6 | 0.2494 |
| >=50% Hispanic | 36,236 | 14.0 | 8,388,109 | 16.5 | 369 | 3.0 | 37.2 | 34.5 | 40.1 | -15.9 | -19.2 | -12.6 | <.0001 |
| >=50% Other races | 2,289 | 0.9 | 806,065 | 1.6 | 339 | 2.8 | 26.7 | 23.8 | 29.9 | -26.4 | -30.0 | -22.9 | <.0001 |
| >=50%-<90% White | 122,281 | 47.3 | 23,031,224 | 45.4 | 5,003 | 41.0 | 47.0 | 45.5 | 48.5 | -6.1 | -8.5 | -3.8 | <.0001 |
| >=90% White | 34,395 | 13.3 | 6,083,070 | 12.0 | 5,475 | 44.9 | 53.1 | 51.2 | 55.1 | 0.0 | [reference] | | |

a. Group 1 excludes Arkansas, Colorado, Florida, Idaho, Maine, Montana, New Mexico, New York, North Dakota, Mississippi, Missouri, Nebraska, Pennsylvania, Tennessee
b. Group 2 excludes Georgia, Illinois, New York, Pennsylvania, Texas, Utah
c. Group 3 excludes Arkansas, Colorado, Florida, Georgia, Illinois, Idaho, Maine, Montana, New Mexico, New York, North Dakota, Mississippi, Missouri, Nebraska, Pennsylvania, Tennessee, Texas, Utah

**Fig. S2. States excluded in sensitivity analysis due to potential data completeness and quality issues**

Division Groupings provided for reference
